# Ghana’s Journey towards Universal Health Coverage: The Role of the National Health Insurance Scheme

**DOI:** 10.3390/ejihpe10010009

**Published:** 2019-10-01

**Authors:** Daniel Dramani Kipo-Sunyehzi, Martin Amogre Ayanore, Daniel Kweku Dzidzonu, Yakubu AyalsumaYakubu

**Affiliations:** 1 Legon Centre for International Affairs and Diplomacy, College of Humanities, University of Ghana, P.O. Box LG 25, Accra, Ghana; 2 Department of Health Policy, Planning and Management, School of Public Health, University of Health and Allied Sciences, PMB 31 Hohoe, Ghana; mayanore@uhas.edu.gh (M.A.A.); ddzidzonu@uhas.edu.gh (D.K.D.); 3 Department of Epidemiology and Biostatistics, School of Public Health, University of Health and Allied Sciences, PMB 31 Hohoe, Ghana; yyakubu@uhas.edu.gh

**Keywords:** national health insurance scheme, exempt groups, universal health coverage, Ghana

## Abstract

Background: the main aim of the study is to find if the National Health Insurance Scheme (NHIS) in Ghana is achieving universal health coverage (UHC) or not. The study gives the trajectories of health policies in Ghana and their implications on long term health financing. NHIS in Ghana was implemented in 2004, with the aim of increasing subscribers’ access to health care services and reduce financial barriers to health care. On equity access to healthcare, it addresses two core concerns: (1) enrolling particular groups (persons exempted from annual premium payments) and (2) achieving UHC for all citizens and persons with legal residence. It utilizes a multifactor approach to the conceptualization of UHC. The research question: *Is Ghana’s NHIS on course to deliver or achieve universal health coverage*? Methods: we used qualitative methods. In doing so, the study engaged participants in in-depth interviews, focus group discussions and direct observations of participants in their natural settings, like hospitals, clinics, offices and homes, with purposive and snowball techniques. This data triangulation approach aims to increase the reliability and validity of findings. Results: the empirical evidence shows NHIS performed relatively well in enrolling more exempt groups (particular groups) than enrolling all persons in Ghana (UHC). The biggest challenge for the implementation of NHIS from the perspectives of health insurance officials is inadequate funding. The health insurance beneficiaries complained of delays during registrations and renewals. They also complained of poor attitude of some health insurance officials and health workers at facilities. Conclusions: both health insurance officials and beneficiaries emphasized the need for increased public education and for implementers to adopt a friendly attitude towards clients. To move towards achieving UHC, there is a need to redesign the policy, to move it from current voluntary contributions, to adopt a broad tax-based approach to cover all citizens and persons with legal residence in Ghana. Also, to adopt a flexible premium payment system (specifically ‘payments by installation’ or ‘part payments’) and widen the scope of exempt groups as a way of enrolling more into the NHIS.

## 1. Background

Ghana gained her political independence in 1957 and has since assumed her own public policies, including health policies. Ghana’s health policy at independence was ‘free health care for all’. The implementation process of ‘free health care for all’ was limited to public health facilities (hospitals, clinics) with less involvement of private health facilities, especially private for-profit facilities [1,2]. The poor collaboration between public and private facilities in the provision of health care services was more serious in rural Ghana where few private health facilities were licensed to provide essential health care services. This situation exposed the rural folks in Ghana to untrained and unlicensed service providers which affected accessibility and quality control [2]. Ghana’s health system at independence was highly centralized, and this also affected rural areas, as health services were far from them.

Ghana’s ‘free health care for all’ was not sustainable by successive governments. The military rulers who overthrew the first president, Kwame Nkrumah, abolished the free health care and replaced it with ‘user-fees’. This meant that people must pay for the cost of some health care services and drugs at health service delivery points. The ‘user-fees’ started in 1969 under the Hospital Fees Decree which later amended to Hospital Fees Act in 1971. Most Ghanaians and persons’ resident in Ghana complained of the ‘user-fees’ as unfriendly and that they did not promote equity in health care services. The reason being that the poor and vulnerable people had limited access to health care services and essential drugs [3,4,5,6].

By 1985, Ghana’s health system changed from ‘user-fees’ system to ‘cash and carry system’ (CCS). The CCS is a practice where people must pay cash before they are attended to at health facilities (hospital, clinics, and others). The ‘cash and carry system’ created more health inequities among various groups, rich and the poor, rural and urban, workers and unemployed groups. The rich and privileged groups could pay, and thus had more access to health services than the poor and vulnerable groups [6,7]. 

The first remedy to health inequalities is the establishment of Community-Based Health Insurance Schemes (CBHISs) in Ghana. The first most successful CBHISs took place in Nkoranza in 1992, the second took place in West Gonja Hospital in Damongo in 1996, as initiatives of the Catholic Church. Besides the two, there were other CBHISs in the 1990s in Ghana [8,9]. The CBHISs were faced with geographic limitations since the majority of the Ghanaian population were left out of CBHISs. The dilemma with which the Government of Ghana was faced in the 1990s was how to effectively address the inequity gap between the rich and poor, between rural and urban dwellers and between communities covered with CBHISs and those without insurance coverage. 

This inequity situation compelled the government to seek alternative health policy which would be more accessible to all the citizens and other persons resident in Ghana and to improve health care services in Ghana. This led to the introduction of the National Health Insurance Scheme (NHIS) in Ghana in 2003, which was implemented in 2004 [10,11]. 

The crux of the matter is that Ghana needs a health policy that will adequately address the gap between the rich and the poor in terms of access to healthcare services at health facilities across Ghana. For this reason, this study examines the efforts made so far by the NHIS towards achieving UHC, since its implementation in 2004. 

Table 1 illustrates the trajectories of health care policies in Ghana from independence (1957) to the introduction of NHIS and their implications.

## 2. Universal Health Coverage (UHC): What Does It Mean?

Universal health coverage is defined, or conceptualized, in the following terms as ‘access to adequate health care for all at an affordable price’, ‘income and risk cross-subsidies in health systems’, securing ‘promotive, preventive, curative and rehabilitative services’ at an affordable cost, and it is about the people having access to key healthcare services without ‘physical and financial barriers’ or financial hindrance. Thus, UHC is about the percentage of population covered, the number of health services covered, be it general and or specific services [12,13,14,15]. 

## 3. Strategies towards Achieving UHC

**Figure 1:**
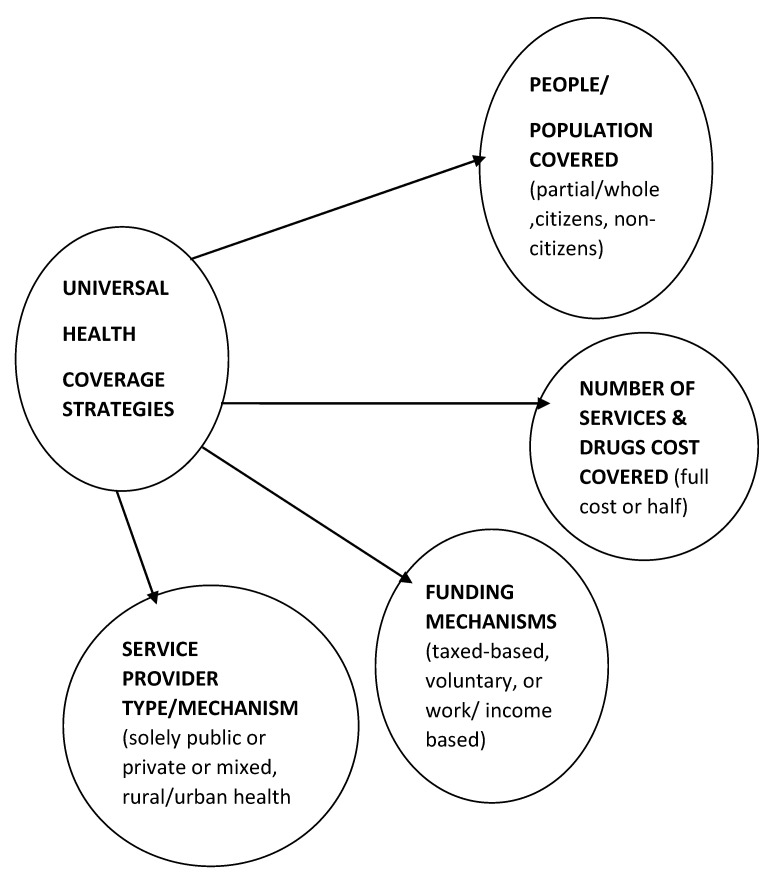
Pathways or strategies towards achieving universal health coverage.

One key strategy towards achieving UHC is the adoption of a ‘pro-poor pathway’ interventions or policies. Those states hold the view that in investing in UHC, its ‘economic benefits’ outweigh the costs [16]. In this strategy, states must set their priorities right, have comprehensive services to be covered, have a drug list and have some guidelines for the delivery of health care services. Another strategy moving towards UHC is to consider ‘fairness and equity’. These are two key elements to be factored in national policies [16]. Other strategies that may be incorporated into national health policy towards achieving UHC are access to health care services without financial hardships, apply progressive financing, or regressive financing, proportional financing, pro-poor or pro-rich distribution of benefits [15,16]. Other scholars identified three strategies towards UHC: ‘who is covered’ (persons), ‘what is covered’ (health services) and ‘proportion of cost covered’ [13]. The literature above suggests there are various ways countries adopt towards UHC. Figure 1 shows some of the strategies some countries adopt towards achieving UHC.

### Analytical Framework

In Figure 1 and in literature are shown some strategies, such as general taxed revenue, where some health care services and drugs are covered, with nationwide coverage, where the health system recognizes public and private health providers to ensure equitable access to healthcare services for health insurance beneficiaries or clients [13,14,15,16].

## 4. Ghana’s National Health Insurance Scheme Pathway Towards UHC

Ghana’s NHIS first step towards UHC was the district-wide schemes which transitioned into a nation-wide scheme. For sustenance and smooth operation of NHIS, a National Health Insurance Fund (NHIF) was established by an Act of Parliament of Republic of Ghana under Act 650 [17]. Act 650 passed in 2003 and was amended with Act 852 in 2012 [18,19]. NHIS funding sources as in ‘section 78’ include: (a) a health insurance levy; (b) 2.5% of workers 17.5% contribution to the Social Security and Pensions Scheme Fund; (c) money allocated to NHIF by Parliament; (d) money that accrues to NHIF from investments made by the NHIS Council; and (e) ‘grants, donations, gifts’ and other ‘voluntary contributions’ [17]. 

The membership of NHIS is open to all citizens and persons resident in Ghana [17,18]. Members who contribute to the Social Security and National Insurance Trust (SSNIT) are registered without payment of an annual premium, while non-social security contributors are to pay an annual premium. Initially, the annual premium varied from one district to another. In Tamale Metropolis (the study area), the annual premium was fourteen Ghana Cedis (GH₵ 14.00 = US$ 3.66492) (with currency converter on 17.04.15). Currently, as of 2019, the annual premium is the same everywhere in Ghana and the new registration is 30 Ghana Cedis (GH₵ 30.00 = US$ 5.48592) for persons in the informal sector from 18–69 years old (with currency converter on 07.09.19). Formal sector workers (SSNIT contributors) and informal sector workers (premium payees) are the main contributors of NHIS in Ghana [17,18,19].

On NHIS provision on ‘exempt groups’ (categories of persons in Ghana exempted from payment of annual premiums), this is what National Health Insurance Regulation 56 stipulate ‘*(a) persons under eighteen years of age and both of whose parents or guardians are contributors; (b) under eighteen years of age and whose parent or guardian has been proven by the scheme to be a single parent or guardian; (c) a pensioner under the SSNIT Scheme; or (d) seventy years or over seventy years of age’* [18]. 

Another category of persons exempted from payment of annual premium is the core poor in Ghanaian society (‘indigent persons’). On enrolling indigent persons, Regulation 58 provides a ‘means test’. The ‘means test’ stipulates a person shall be deemed or classified as indigent under these conditions: ‘*(a) is unemployed and has no visible source of income; (b) does not have a fixed place of residence according to standards determined by the scheme; (c) does not live with a person who is employed and who has a fixed place of residence; and (d) does not have any identifiable consistent support from another person’* [18]. All district schemes are to identify poor (indigent persons) and submit such names to the NHIS Council for ‘validation’ (approval). Some flexibility has been added to the ‘means test’ by the new Act, 2012.

However, when the district schemes list of indigent persons exceeds 1.5% of the total membership of the scheme, the NHIS Council (national) has the power to verify lists submitted by district schemes by ‘whatever means the Council determines’. The persons displeased can complain to the district schemes Complaint Committee [18]. Other exempt groups include the aged (70 years and above), who are exempted from payment of annual premium once proven by scheme officials. The last, but not the least, category is pregnant women. They were added to exempt groups by an ‘executive order’ from the presidency in 2008 after the passage of the National Health Insurance Act in 2003. This addition to pregnant women, it was necessary to prevent, or reduce, maternal deaths in Ghana. The main criterion for the selection of pregnant women is that a woman is ‘certified’ as pregnant by a qualified medical officer. Table 2 shows the categories of exempt groups under NHIS in Ghana.

**Figure 2:**
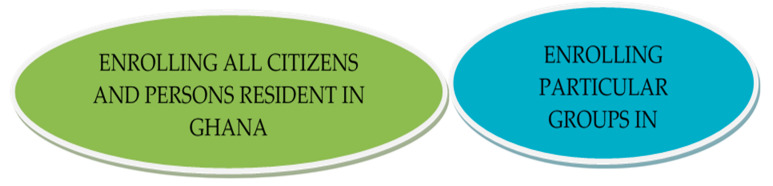
National Health Insurance Scheme (NHIS) enrolment strategy for all persons and exempt groups in Ghana.

The second strategy adopted by NHIS towards UHC is in its enrolment strategies. Thus, NHIS targeted two categories of persons: by enrolling all persons and the particular groups as in Figure 2. Figure 2 shows the two main enrolment strategies of the NHIS in Ghana.

Figure 2 shows that Ghana’s NHIS combines enrolment of all persons (universal provision) with an enrolment of categories of persons in Ghanaian society (particular provision). This suggests that NHIS in Ghana is reconciling universal and the particular groups provision towards health equity meeting the healthcare needs of all persons in Ghana as well as the ‘vulnerable or disadvantaged’ groups in society. This supports the argument that a contemporary social policy adopts a mixed provision [20].

## 5. Methods 

A qualitative research method was used in the study. Four sources of data collection methods were adopted. We used in-depth interviews and focus group discussions (FGDs), direct observations and obtained a lot of documents in the study. Persons with NHIS cards and active members were included, while those without NHIS cards and non-members were excluded from the study. The two categories of persons were NHIS implementers and beneficiaries. The implementers were the National Health Insurance Authority (NHIA) staff while the beneficiaries were NHIS members. We used two sets of interview guide questions, one for implementers, the other for the beneficiaries. The implementers were selected based on the positions they occupied in relation to the implementation of NHIS. On the part of NHIS beneficiaries’, we were mindful to interview persons based on their categories as either contributors or exempt group members. We adopted ‘purposive sampling’ technique in order not to leave out any of the categories of beneficiaries [21,22]. This technique was more appropriate than a simple random sampling method. Another method used in data collection was the ‘snowball technique’ [23]. Interviews with one person led to another, which helped in reaching out to persons we initially did not identify. We conducted about 30–60-minute interviews, depending on the time the respondent made available. Interviews enabled beneficiaries and implementers to share their views and experiences on NHIS. In addition, we made good use of what some researchers called on-site ‘direct observations’ [23,24]. This method helped us to observe directly at facilities and health insurance offices what happened between the implementers and beneficiaries during health service provision to health insurance clients. The direct observation method helped us to uncover certain things interviews could not provide. As a result, certain practices and actions were noted, which could not be said during interviews. Moreover, we made extensive use of documents, thus making ‘textual meaning’ from the data collected as social science researchers encourage the use of documents [21,22,23,24]. Relevant documents, like annual health insurance reports at district, regional and national levels to examine the coverage of NHIS in Ghana, were used. Beside the one-on-one interviews, we also utilized focus groups discussions (FDGs) among beneficiaries at health facilities. Some interviews took place outside health facilities and NHIA offices. Some locations were selected in Tamale based on the socio–economic and income levels based on the 2010 Population and Housing Census [25]. The three phases were done for 12 months as part of a larger project, but not only for this publication. Validity strategies were used, such as ‘member-checking’, where participants were given the opportunity to determine the accuracy of fieldwork information obtained earlier and for the ‘rich, thick description’ of study findings. These strategies were possible due to the prolonged stay in the field. These explained the rationale for the three phases of data collection [21,22]. We addressed these ethical issues by seeking for institutional permissions from the Ghana Health Service (GHS) and the NHIA. Respondents informed consent was obtained while other issues of privacy, anonymity, and confidentiality were adhered to in the study [21,22,23,24].

We adopted an in-depth case study approach on ‘contemporary events’ [26,27]. The contemporary event refers to the implementation of Ghana’s health insurance scheme towards achieving UHC. The study involved ‘participants in their natural settings’—health facilities, offices and at their homes [26,27]. Our choice of case study approach was for the purpose of in-depth analysis of the phenomenon and for analytical generalization [26,27,28]. In-depth interviews focus groups discussions (not more than seven persons), on-site direct observations and review of documents were done. The study utilized data triangulation, which was used for the reliability and validity of the study findings [29,30]. Where we found differences from interview sources, we used observations as a check. This additional method was a necessary measure for the reliability of the interview responses or findings as we matched data obtained from interviews with observations as well as we matched some interview responses with document sources. These were measures we adopted towards the validity and reliability of findings.

The study took place in the Tamale Metropolis of Ghana. The rationale for Tamale Metropolis was due to its population size as the biggest city in Northern Ghana, and also, as the city with the largest number of NHIS accredited facilities in the north. The data analysis involved the following: coding and content analysis along with some occurring patterns, with major themes like NHIS coverage for all persons and the particular groups’, sub-themes like behavior, attitudes of staff (implementers) and beneficiaries among others. The interviews voice recordings were stored on hard drives. The field notes were also typed and analyzed (meaning out of the text). Interviews were audio-recorded and transcribed by professional language translators, some translations were from local languages like Dagbani, Gonja, Twi to the English language. Participation was voluntary, anonymity, the privacy of respondents, translators were guaranteed [31]. Table 3 shows the profile of respondents. 

## 6. Results

The results focused on NHIS coverage for all persons in Ghana, including citizens and other persons resident in Ghana. This section also presents results on NHIS coverage for particular groups, some prospects made towards achieving UHC and highlight some key implementation challenges.

### 6.1. NHIS National Coverage for All Citizens and Persons Resident in Ghana

Governmental document sources revealed an increasing trend of membership since its inception in 2004 [32,33,34,35,36]. However, the Oxfam report indicated NHIS coverage in Ghana has been ‘exaggerated and could be as low as 18%’ coverage for the entire country [11,37]. 

The NHIA made some changes to its method for calculating NHIS coverage from 2010 onwards. The new percentage coverage is based on active membership (new registration and renewals) but not on the aggregate figure of the previous year. The baseline for calculation of the active membership of NHIS is the 2010 Population and Housing Census with a population of Ghana as 24,658,823 [25]. The percentage coverage of NHIS in Ghana in this study starts in 2010 as shown in Table 4.

Data from Table 4 shows a slowly increasing trend in membership of NHIS in Ghana from 2010 to 2013. It was only from 2012 to 2013 that experienced a higher increase in percentage coverage. The year 2013 marked ten (10) years of implementation of NHIS, where 38% of Ghana’s population is covered. 

Furthermore, not much increase occurred in 2014. In 2015 the national percentage coverage for NHIS stood at 38.4% [38]. This has been the scheme’s highest growth stage since its implementation in 2004. These statistics on coverage of NHIS in Ghana is an indication that Ghana had made progress towards UHC of 38.4% within ten years of implementation of NHIS (2004–2013).

### 6.2. NHIS National Coverage for Particular Groups 

In this section, we examine the percentage coverage for the categories of NHIS beneficiaries. The aim is to ascertain which category of beneficiaries are enrolled more into the NHIS in Ghana and possibly look at the factors that accounted for such differences in their levels of enrolment. 

The NHIS has performed relatively well in increasing numbers of exempt groups’ enrolment into the insurance scheme. Documents or records obtained from the NHIA show an increasing number of exempt groups especially children and persons below 18 years. Children and persons below 18 years have the highest coverage among exempt groups. This is followed by pregnant women. The least exempt groups’ members are the indigent persons (core poor in Ghanaian society) and the aged 70 years and above between 2010 and 2012 [32,33,34,35]. Reasons for the increasing trend in the membership of exempt groups may be partly due to their non-payment of NHIS annual premium among other conditions. Since the health insurance law excludes exempt groups from payments of the annual premium. The exemption provision may explain the increasing trend for the years.

**Figure 3:**
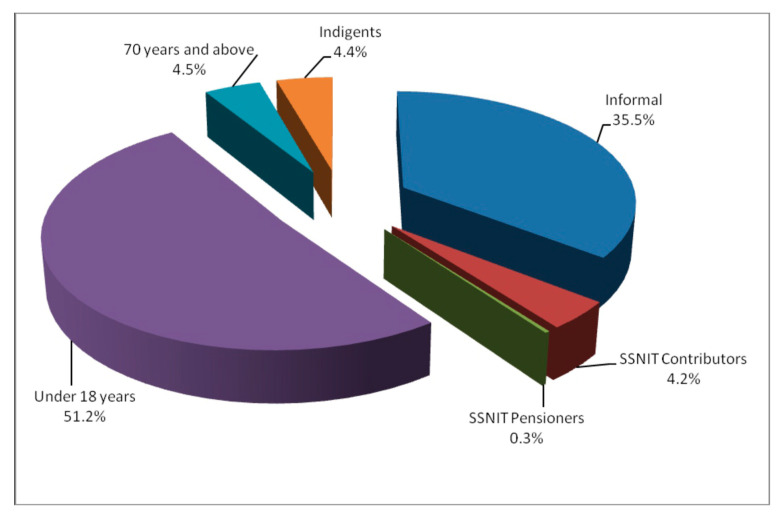
Active NHIS Members by categories in 2012. Source: adapted from the National Health Insurance Authority Annual Report (2012, p. 20).

We also look at the percentage coverage for persons who make direct contributions or payments to NHIS. The two main contributors to NHIS are informal and formal sector workers. The informal sector workers contribute more than the formal sector workers (SSNIT contributors). Figure 3 depicts the categories of NHIS members in 2012.

In Figure 3, NHIS provision on meeting the health needs of particular persons (exempt groups) is shown to be positive and progressive, as documents records show 51.2% coverage for children and persons less than 18 years. This is an indication that more persons belonging to this particular group are covered by NHIS and have access to health care services across Ghana. The second exempt group with more percentage coverage is the aged (70 years and above) with 4.5%, followed closely by indigent persons with 4.4% and SSNIT pensioners as the least exempt group with 0.3% percentage coverage as at 2012. This data shows 64.6% of persons who enrolled in NHIS are exempt groups (persons who do not pay an annual premium or make a direct contribution to NHIS). Thus, most members of NHIS in Ghana are exempt group members [32,33,34,35]. 

Additional data obtained from the National Health Insurance Authority at the end of 2014 shows that the percentage coverage for exempt group members was 65.7% [38]. This is the composition of the categories of exempt groups coverage in 2014: children and under 18 years with 44.8%; followed by indigents (core poor) with 14.5%; the least exempt group being SSNIT Pensioners with 0.2% coverage. The 2014 data also show that total contributors constituted 34.3% (SSNIT contributors with 3.6% and informal sector workers with 30.7%). NHIA percentage coverage as of May 2015 shows less than 40% of Ghana’s population is covered by NHIS, more efforts are made to increase coverage [38].

## 7. Some Implementation Prospects and Challenges towards Achieving UHC in Ghana

### 7.1. Payment of NHIS Annual Premium (Perspectives of Contributors)

On the question of affordability of annual premium by contributors, two interviewees indicated the amount was high while eight of them said the amount was affordable. The two respondents said the amount of GH₵ 14.00 US$ 3.7 for the registration fee and annual premium was high and not affordable. The two participants indicated that there was a need for the payment of annual premium by instalments. This was what one health insurance premium payee said:

*You see these insurance people are charging us too much, they think it is easy to get 14 Ghana cedis so easily to be paying every year. Please tell them to reduce the insurance fee or make it possible for us to pay the amount small, small or better still make it free for all.*

Majority of NHIS contributors that we interviewed (eight out of 10) answered in the affirmative that the amount of premium was quite low and manageable. Some compared annual premium to cost of hospitals and clinics attendance cards and indicated that most health facilities attendance cards were more expensive than the NHIS annual premium. Most premium payees disagreed that the annual health insurance premium is too high. Thus, the premium is affordable and manageable.

This was how another contributor (premium payee) commented on the NHIS annual premium:

*My bro just take a taxi from Fuo to Tamale Teaching Hospital and back and see how much you will pay and compare that with what you will pay for the whole year on health insurance and then you will see that you are just paying nothing, I say nothing.*

An SSNIT contributor also expressed her opinion on the annual premium:

*Even though I don’t pay cash for the annual premium because it is deducted from my salary at the controller and accountant general department in Accra, I believe the amount is too small compared to the cost of treatment, drugs, diagnoses which are all covered by health insurance scheme for free. So, this premium thing is just a penny.*

The responses we obtained from in-depth interviews on the issues of affordability or otherwise of the annual premium showed a mixed feeling among NHIS contributors. Some said the amount was high (minority view—two participants), most said the amount was affordable and manageable (majority view—eight participants). These are the perspectives of contributors of NHIS at the local level.

### 7.2. Perspectives of Exempt Groups 

Exempt groups are the categories of persons exempted from payment of the NHIS annual premium. For the exempt groups, the questions were not on affordability or otherwise of the annual premium, but the questions focused on issues around registration and renewals of membership of NHIS in Ghana. The aim is to get or solicit their views on the processes, procedures and to share their experiences.

For children and persons below 18 years, results show that most children were registered by parents or guardians. Those more than 10 years were able to speak with the consent of their parents or guardians. The teenagers who could read and write answered the questions themselves, while those who could not read and or write, answered the questions verbally. With the consent of their parents or guardians, we recorded their responses in the field. On the amount paid as registration fees, one could not tell the amount paid, the other said he did not notice what was paid by his guardian in the health insurance office in Tamale while the third stated that his mother paid GH₵ 5. We asked further if he was sure of the amount the parent paid, he repeated:

*My mother paid five Ghana cedis so health insurance is not free for children.*

We probed with the parent who confirmed that the registration fee is five Ghana cedis (GH₵ 5). She added that when she gave the health insurance registration officials the five Ghana cedis (GH₵ 5), they took the whole amount. The parent response collaborated with the less than 18 years of an exempt group member on registration fees at the health insurance office in Tamale. We solicited the views of health insurance officials to ascertain the amount paid as registration fees for exempt groups in Ghana. We then sought clarity on this issue from a local level health insurance official at the scheme office in Tamale Metropolis. The health insurance officials we interviewed indicated that children and those below 18 years do not pay for annual premium and that they are to pay only registration fees. This was what the official at Public Relation Unit said:

*Normally people get confused with registration fees and annual premium, I want to put it clearly, all exempt groups I think you know they are to be registered free of charge. However, they are expected to pay for registration fee which is only two Ghana cedis.*

The response confirmed that the registration fees are two cedis (GH₵2) but not five cedis (GH₵5). The views expressed by the two persons below 18 years and health insurance official show that the exemption policy is implemented at the local level where children and persons below 18 years pay for only registration fees while being exempted from payments of the annual premium. 

Pregnant women: five pregnant women we interviewed confirmed they did not pay for an annual premium. But they paid different prices as registration fees. Out of the five pregnant women interviewed, two said they paid two cedis (GH₵ 2) while three said they paid five cedis (GH₵ 5) as registration fees. Health insurance officer in Tamale Metropolis office has this to say on some strategies the scheme adopted to get more persons enrolled into NHIS towards achieving UHC:

*Our aim is to get more people to enroll into the national health insurance scheme, so we have established a number of health insurance communities across the metropolis in order to reach out with people, the registration fee is GH₵ 2 and the annual premium is GH₵ 12 with total GH₵ 14.*

One of the strategies towards UHC is the establishment of health insurance communities across Ghana. Each district scheme office is divided into zones known as health insurance communities as a way of reaching out with the Ghanaian population (people) to register for NHIS in Ghana. Also, the response of the NHIS official at the scheme office confirmed that the registration fee is two cedis. On differences on registration fees, we made some direct observations at the scheme office. We noted that NHIS beneficiaries paid a different amount (prices) to officials’ in-charge of registration. We observed that some paid GH₵ 5 while others paid less than GH₵ 5. In some cases, the registration officials were not able to get smaller cedis denominations. But these were exceptional cases on the issue of registration fees. It is worthy to note that the difference of three (GHS 3) is quite too high, given the financial status of the people at the local level. Such differences in registration fees could be a disincentive to enroll more exempt groups into the NHIS. The registration fee was not documented, as we tried to get the exact amount from the annual reports and other records we obtained from the field. Thus, we relied on what the officials said.

Indigent Persons (poor in society): two indigent persons were identified by the health insurance scheme and registered free while one indigent had a different view on how he was registered. This was what the indigent (poor) said at one focus group discussion (FGD) meeting:

*One man came around my house and saw me and ask me if I am registered for health insurance I said no and he sent me to the office to register me. Now I don’t have anybody to register me.*

We solicited the views of other indigents and this was how another indigent said in an interview: *I was registered by an NGO for three years now, they always register me*.

The responses above confirmed the existence of an exemption policy, where some particular persons, like the indigents, are exempted from payment of annual premiums. But the empirical evidence we obtained from in-depth interviews, focus group interviews and documents showed that there are serious problems or challenges in identifying indigent persons at the local level particularly in the strict application of a means test in line with the NHIS law passed in 2003 and regulations. By application of the means test, an indigent person is the person that is unemployed, has no source of income, no fixed place of residence and does not receive support from any person [17,18]. But the challenge in the application of the means test had changed with the new Act (852), 2012 that added more vulnerable persons in the streets, orphanages, and the inclusion of Livelihood Empowerment Against Poverty (LEAP) beneficiaries among other social interventions programs in Ghana.

On the application of the ‘means test’, this is what health insurance scheme official said:

*My brother in this Tamale how will you be able to track people earnings especially those in the informal sector, where people sleep, the kind of support they get from their family members and other persons. These are real challenges, so we work hand in hand with community leaders to help us identify the very poor ones and to get them to enroll in a health insurance scheme.*

The in-depth interviews responses and FGDs with beneficiaries and health insurance officials revealed some challenges in getting exempt groups registered for NHIS especially indigents (poor).

Aged (70 years and above) and SSNIT Pensioners: our interviews and interactions with the aged (70 years and above), SSNIT pensioners revealed fewer challenges in identifying and registering them. Data on SSNIT pensioners is available at the SSNIT office, thus there are a few challenges with aged groups largely from the informal sector as compared with identifying the indigent persons.

On renewals of membership of the NHIS among beneficiaries, most beneficiaries indicated that the renewal process is not as cumbersome as the registration process. But, the problem most of the beneficiaries mentioned have is long queues in the health insurance offices. This appeared to be a national problem, particularly in urban centers, where hundreds of beneficiaries turn up to have their ID cards renewed manually at health insurance offices across Ghana. In 2018, the National Health Insurance Authority (NHIA) introduced electronic renewal system. This innovation has solved the problems of long queues, many waiting hours and the congestions at scheme offices. Moreover, the innovative renewal process is to dial *929# under the ‘NHIS Mobile Membership Renewal and Authentication Project’. It is a mobile money payment system which aims at making the renewal process quite easy and to attract more people into the NHIS towards UHC. Table 5 summarizes key prospects and challenges in the implementation of NHIS towards UHC.

## 8. Discussion 

The evidence or field data shows NHIS has contributed positively towards increasing particular groups’ access to health care services in Ghana due to their large enrolment numbers into NHIS. Ghana’s NHIS made progress by providing an insurance cover for over 60% of exempt groups. Thus, NHIS is successful in terms of enrolling and meeting exempt group health needs in Ghana, but more efforts are needed towards achieving its core goal of UHC for all citizens and all persons with legal residence in Ghana. Ghana, as a developing country is still faced with enormous financial challenges in attaining UHC for all persons (citizens and persons with legal residence). Just as other African countries are also making efforts towards UHC. Ghana reached 38% nationwide coverage from 2004–2015, Kenya with 11% formal sector, 1.3% at community-based schemes, Rwanda from 1994–2016 at 80% with informal and community-based schemes. Others like Tanzania from 2001 with 17% formal sector and 4% informal sector, Ethiopia community-based schemes from 2010/2011 at 7.5%. All these African countries are all making efforts, pathways towards achieving UHC [39]. Beyond Africa, there is the success story of Japan achievement of UHC in 1961. Other Asian countries, like Thailand, Turkey, Indonesia, Vietnam, are making efforts towards UHC, likewise some South American countries like Brazil, Peru. All these countries exhibit diverse or different economic, geographic and political contexts towards UHC. Bangladesh/Ethiopia as starters; middle-level progress Ghana, Indonesia, Peru, and Vietnam; Brazil, Thailand, and Turkey have achieved UHC, yet more efforts to improve quality of services and financial protection; Japan and France achieved UHC with comprehensive coverage and effective financial protection. There are indeed good lessons on the success stories and opportunities for those countries who achieved UHC while there are some lessons on the challenges towards UHC [40].

It is also important to understand and appreciate that Ghana’s NHIS is not a general tax-based health insurance system as happened in most developed European countries such as Denmark, Norway, Sweden, Finland, Germany, France, Britain among others. But Ghana’s NHIS system is rather based on voluntary health insurance system where members must pay an annual premium to register and make efforts to renew their NHIS membership every year [17,18,19]. This is a double burden on the NHIS beneficiaries in Ghana to register and renew membership every year, while beneficiaries of health insurance systems in developed Europe are free from such a double burden. Hence, the voluntary nature of NHIS is a key contributory factor for the low enrolment percentage coverage for all citizens and other persons resident in Ghana into NHIS. 

Interviews and document records show NHIS is not enrolling many of the core poor (indigents) in society into NHIS as the core poor in society constitute one of the least numerous members of the NHIS (see Figure 3). This is not good news for the NHIS in terms of poverty reduction among the poor in Ghanaian society. This study finding concurs with other studies findings that the NHIS is not a ‘pro-poor’ social policy in Ghana [36,37,41]. This study agrees with these findings that Ghana’s NHIS is not enrolment more of the core poor people in Ghanaian society and this remains a big challenge. Hence, pragmatic steps must be taken by health insurance authority to enroll more of the poor, more collaboration with other institutions like social work department, Ministry of Gender and Social Protection to increase LEAP beneficiaries, work with other non-governmental organizations, community leaders and other civil society groups to enroll more of the poor and vulnerable groups into NHIS.

Despite NHIS efforts towards achieving universal health coverage (health care provision for all persons) in Ghana, this study found that most of Ghanaians (over 60%) are left out of NHIS at the national level. This study finding concurs with the findings of Averill that only a quarter of Ghanaian population is covered by NHIS while most of the population continue to make ‘out-of-pocket payments to access healthcare’ in Ghana [37]. This is not good for NHIS move towards UHC (enrolling all citizens and persons with legal residency into the NHIS). More efforts to enroll more people are crucial.

## 9. Conclusions

The voluntary nature of the NHIS is identified as a key inhibiting factor towards achieving UHC. The government of Ghana should make efforts to broaden the tax-based to generate enough revenue to cover all citizens and persons in Ghana. 

Ghana’s NHIS exhibits universalism with particularism. The NHIS has performed relatively well in its particularistic provision (enrolling exempt groups to meet the health needs of exempt group members) more than universalism provision (enrolling all persons to meet the health needs of all people). The increased exempt groups over NHIS contributors have financial implications for the implementation of the NHIS. The more exempt groups are enrolled into NHIS, the more the government would have to transfer more money into NHIS fund to be used as subsidies for exempt groups and for reinsurance. 

Further studies should examine the implementation of NHIS on universalism and particularism principles, specifically to look at the nature and the kind of healthcare services rendered to NHIS beneficiaries across Ghana. Also, further studies should make a comparative study of Northern and Southern Ghana on the implementation of the NHIS. Furthermore, quantitative studies should be conducted in mapping the numbers of exempt groups with contributors in terms of percentage coverage from the inception of NHIS to date. The last but not the least is the need to thoroughly investigate into the inhibiting factors militating against Ghana’s NHIS move towards achieving UHC particularly along with these contexts: social, economic, psychological and political factors.

## 10. Implications of Study Findings to Health Policy Planners or Makers

This study identifies a key factor inhibiting against the achievement of UHC. This factor is the design of Ghana’s national health insurance scheme (health policy). The health policymakers choice of voluntary membership of NHIS made it difficult, if not impossible, for the national health insurance scheme (health policy) to achieve UHC. The study recommends that there is a need to review the design and possibly redesign it towards a compulsory membership based on general tax revenue in Ghana. This tax-base should be broadened to cover all public and private sector actors (workers). In such a broad tax system, free riders must not have a leeway to operate.

### 10.1. Implications for the Public

The Ghanaian public needs to support the NHIS. In doing so, they should change their negative attitudes into positively motivated attitudes and actions to help reduce the abuses on NHIS. This is an effective way of curbing the abuses at the facility level (hospitals and clinics) and at the scheme level (health insurance offices) across Ghana. The government should desist from the politicization of NHIS in terms of appointments and disbursement of NHIS funds. In times of crisis, we need an all hands-on deck approach to address the problem or challenge, instead of the political blame game.

### 10.2. Limitations and Future Research

The study is largely qualitative with the possible challenge of generalization of findings. Thus, future research should focus on a statistical analysis of various groups on the regional or rural-urban basis.

## Figures and Tables

**Table 1:** Health policy reforms in Ghana.

Year	Policy Type	Ownership	Funding	Benefits	Coverage	Challenges
**1957**	Free health care for all	Public Health Facilities	Public	Free healthcare Services for all	Nation-wide	Sustainability Problem Limited to the Public sector
**1969**	User-fees	Public and Private Facilities	Partly Public and Individual Payments	Sustainable Favorable to Rich People	Nation-wide	Inequity Access to Healthcare Services
**1985**	Cash and Carry System (CCS)	Public and Private Health Facilities	Cost-Sharing and Cash Payments	Cost recovery, More revenue, Prevent wastage, Check clients frequent visits	Nation-wide	Inequity access, High Mortality at Homes for fear of facilities visits of CCS
**1992**	CBHIS	Private Non-profit Facilities	Privately Funded	Provide health Insurance Cover for the locality	Community Based	Geographic Limitations
**2004**	NHIS	Public-Private	Partly Public and Individual Payments	Comprehen-sive package of 95% common diseases	Nation-wide	Sustainability Problem, Inadequate Funding

Source: Literature Review [1,2,3,4,5,6,7,8,9,10,11].

**Table 2:** Categories of the National Health Insurance Scheme (NHIS) exempt groups in Ghana.

**Exempt Groups**	Persons Less than 18 Years	Indigent Persons	70 Years and above	SSNIT Pensioners	Pregnant Women
**Description**	Children, <18	Poor ones	The aged	Retired Workers	Certified Pregnancy

Source: [17,18,19].

**Table 3:** Categories of Participants (Interviewees).

Beneficiaries	Number	The Staff of Health Insurance	Number	Grand Total
Premium Payees	6	NHIA National Director	1	
SSNIT Contributors	4	NHIA Regional Director	1	
Aged (70 years and above)	3	Metropolitan Scheme Official	3	
Children (less than 18 yrs)	3	Public Relation Officer	1	
Pregnant Women	5			
**Total Beneficiaries**	**24**	**Total NHIA officials**	**6**	**30**

Source: Fieldwork Interview Data, 2012; 2013; 2014.

**Table 4:** National coverage of NHIS from 2010–2013.

LEVEL	Active Members of NHIS in Ghana			
	2010	2011	2012	2013
**National**	8,163,714	8,227,823	8,885,757	10,145,196
**Percentage**	33.1	33.4	35.0	38.0

Source: National Health Insurance Authority Annual Reports, 2010–2013.

**Table 5:** Prospects and challenges of Ghana’s NHIS towards achieving universal health coverage (UHC).

KEY PROSPECTS OF GHANA’S NHIS	KEY CHALLENGES OF GHANA’S NHIS
❖ Enrolling More Exempt Groups ❖ Innovations with electronic renewals ❖ Covers about 90% of common diseases ❖ Large benefits package ❖ Increased accessibility to facilities	❖ The problem of voluntary membership ❖ Less coverage for NHIS contributors ❖ Poor attitude of implementing officials ❖ Discouraging long waiting hours ❖ The double burden of registration and renewal

Source: Authors.

## Abbreviations

CBHIS	Community-based Health Insurance Schemes
DMHIS	District-wide Mutual Health Insurance Schemes
SSNIT	Social Security and National Insurance Trust
UHC	Universal health coverage
